# A general framework for classifying costing methods for economic evaluation of health care

**DOI:** 10.1007/s10198-019-01157-9

**Published:** 2020-01-20

**Authors:** Zuzana Špacírová, David Epstein, Leticia García-Mochón, Joan Rovira, Antonio Olry de Labry Lima, Jaime Espín

**Affiliations:** 1 Andalusian School of Public Health/Escuela Andaluza de Salud Pública (EASP), Granada, Spain; 2 University of Granada, Granada, Spain; 3 CIBER en Epidemiología y Salud Pública (CIBERESP), Spain/CIBER of Epidemiology and Public Health (CIBERESP), Madrid, Spain; 4 Instituto de Investigación Biosanitaria ibs, Granada, Spain

**Keywords:** Costing methodology, Economic evaluation, Top-down method, Bottom-up method, Micro-costing, Activity-based costing, A12, I10

## Abstract

According to the most traditional economic evaluation manuals, all “relevant” costs should be included in the economic analysis, taking into account factors such as the patient population, setting, location, year, perspective and time horizon. However, cost information may be designed for other purposes. Health care organisations may lack sophisticated accounting systems and consequently, health economists may be unfamiliar with cost accounting terminology, which may lead to discrepancy in terms used in the economic evaluation literature and management accountancy. This paper identifies new tendencies in costing methodologies in health care and critically comments on each included article. For better clarification of terminology, a pragmatic glossary of terms is proposed. A scoping review of English and Spanish language literature (2005–2018) was conducted to identify new tendencies in costing methodologies in health care. The databases PubMed, Scopus and EconLit were searched. A total of 21 studies were included yielding 43 costing analysis. The most common analysis was top-down micro-costing (49%), followed by top-down gross-costing (37%) and bottom-up micro-costing (14%). Resource data were collected prospectively in 12 top-down studies (32%). Hospital database was the most common way of collection of resource data (44%) in top-down gross-costing studies. In top-down micro-costing studies, the most resource use data collection was the combination of several methods (38%). In general, substantial inconsistencies in the costing methods were found. The convergence of top-down and bottom-up methods may be an important topic in the next decades.

**Electronic supplementary material:**

The online version of this article (10.1007/s10198-019-01157-9) contains supplementary material, which is available to authorized users.

## Introduction

The ultimate task of economic evaluation is the comparison of costs and outcomes of two or more alternatives. This study focuses on the identification, measurement and valuation of resource costs. Most manuals and guidelines recommend to separately report these three steps, which together with some additional procedures, such as discounting and adjusting for risk, is referred to as costing methodology in economic evaluation analysis.

Economic evaluation guidelines state that “all relevant costs” should be included in economic evaluation analysis, irrespective of who bears them [1]. However, it is increasingly accepted that the set of costs to be considered depends on the perspective of the analysis [2], which usually implies that the perspective of a decision-maker restricts the relevant costs to those which are borne or paid for from the budget of the respective decision-maker’s organisation. The “social” perspective is assumed to consider all cost or, at least, the largest or more comprehensive set of resource effects.

Economic evaluation seeks to address a particular research question in which a decision needs to be made, and hence the cost estimates must include all marginal costs of implementing the program [1], taking into account factors such as the patient population, setting, location, year, perspective and time horizon [3]. Health economists need to describe completely and clearly the data and methods used to estimate costs so that users of economic evaluation can assess how appropriate, accurate and precise these methods are likely to be.

However, for diverse reasons, the methods or the reporting of costs for economic evaluation may fall short of these general principles. Cost information routinely produced by health care organisations may have been designed for other purposes, such as meeting financial reporting standards, or for setting tariffs. Health care organisations may lack the sophisticated management accounting systems required to estimate accurate and precise costs of individual services [4]. Cost accounting terminology may be unfamiliar to health economists and this may give rise to a discrepancy between terms used in the economic evaluation literature and established practice in the field of management accountancy. Health economists sometimes make unfounded claims about the theoretical or practical advantages of certain methods (giving rise to generalisations such as “bottom-up micro-costing is generally considered to be gold standard approach for economic evaluation”) [5], which fail to recognise that any cost estimation method requires assumptions or methodological choices and may involve trade-offs or compromises between theoretical soundness and practical feasibility.

More fundamentally, accounting information compiled by health care providers may not always reflect opportunity cost of the resources. This is, in part, a problem of accounting reporting standards, which for public sector providers in some countries are produced on cash flows. Private sector providers working under International Financial Reporting Standards (IFRS) are generally obliged to report accounts on an accrual basis. Some public organisations (such as in the UK) are obliged to undertake full absorption costing as part of their annual financial reporting. Moreover, the concept of “opportunity cost” is subjective and depends on the preferences of the individual that defines it, e.g. spending an hour at the physicians’ waiting room has a different value depending on how busy you are.

To meet these challenges, this study reviews the appropriateness of methods for estimating costs for economic evaluation. To better clarify terminology, we begin with a pragmatic glossary of terms and show how cost accounting methods broadly relate to one another according to a general taxonomy begun by Tan et al. [5]. Building on an existing comprehensive review undertaken in 2005 [6], we undertake a scoping review of recent literature on costing methods, to see how health economists are putting these broad principles into practice. We consider how adequately, completely and clearly each article describes their chosen method, the data and assumptions required to implement the method and how the method addresses criteria of accuracy and precision for economic evaluation.

## Methodology of estimating costs

Costing is time and effort consuming and, therefore, analysts need to decide how accurate and precise the cost estimates need to be. Accuracy refers to whether the estimate is appropriate for the research question (external validity) and the measurement is well founded (internal validity). Precision refers to whether the measurement is reliable (the extent to which repeated measurements under the same conditions would give consistent results), and increases with sample size. Any method for estimating a cost needs to address two broad questions, which will influence the accuracy and precision that can be achieved: (1) the degree of disaggregation used in the identification and measurement of resource and cost components (micro-costing vs. gross-costing) and (2) the method for the valuation of resource and cost components (top-down vs. bottom-up) [1]. Micro-costing identifies resources at very detailed level, while gross-costing identifies resources at a more aggregated level (the distinction is a question of degree). Top-down costing apportions expenditure accumulated at each organisational cost centre down to units of activity, while bottom-up costing first identifies resources used by individual patients and then values these using unit costs to obtain total costs per patient. Using these two dimensions, Tan et al. [5] classified methods into four categories: bottom-up micro-costing, bottom-up gross-costing, top-down micro-costing, and top-down gross-costing [5]. Each of these categories is described in Table 1 using the aforementioned definitions.

**Table 1:** Description of costing methodologies. Source: adapted and completed from 5

		Level and type of data collected	
		Expenditure data collected at organisational level (e.g. cost centre)	Resource use data collected for each individual patient and then multiplied by unit cost to estimate the expenditure
Level of identification of resource use items	Highly detailed resource use items are identified	Top-down micro-costing	Bottom-up micro-costing
	Aggregate resource use items are identified	Top-down gross-costing	Bottom-up gross-costing

## A general framework for classifying costing methods

### Pragmatic glossary of terms used in cost accounting

Table 2 gives a glossary of terms used in cost accounting. The examples and explanations are adapted depending on whether the term is being used for top-down or bottom-up approaches. It should be noted that terminology used for cost-accounting differs from that used for economic evaluation, particularly for the terms “direct” (that are understood in the economic evaluation terms as inputs that are consumed in the provision of health care) and “indirect” cost (that in the economic evaluation terms refers to the effects that a disease has on the affected individual’s productivity).

**Table 2:** Established cost-accounting terminology for top-down and bottom-up methods. Source: Own elaboration. Some definitions are adapted from Atkinson et al. (2001) [7], and from The Accounting Tool Sites (www.accountingtools.com)

Term (and synonyms, if any)	Definition in cost accounting	Explanation and example
General terms		
Direct cost	A cost of a resource or activity that is acquired for or used by a single cost object	An expenditure that can be directly traced in the organisation’s management accounting system to a particular cost object, e.g. a pharmaceutical that is used exclusively for treating a particular diagnosis-related group (DRG) and no other
Indirect cost (also known as variable overheads)	The cost of a resource that is acquired to be used by more than one cost object, but is a variable cost, that is, the quantity used increases with the number of patients treated	E.g. Expenses which are recorded at departmental level which are shared between several patients, such as medical staff or nursing staff
General overhead (also known as fixed overheads)	Expenses which are incurred at organisational level, do not vary with the number of patients treated, and are shared between several departments	E.g. amortisation of buildings, staff training costs, cost of water, electricity and heating
Terms used in top-down costing		
Top-down costing	A costing method where the organisations direct and indirect costs incurred over a given period are assigned to (or “absorbed” by) all the cost objects produced by the organisation	Direct costs are identified directly to cost objects. Indirect costs are “apportioned” to cost objects. In full costing, both variable and fixed overheads will be apportioned to cost objects
Variable top-down costing	A top-down costing method. Organisational direct costs and variable overheads will be assigned to all the cost objects. Fixed overhead costs are not assigned to cost objects	Sometimes used in decisions where the organisations wishes to estimate the marginal cost of its products
Full absorption costing (or full costing)	A top-down costing method. 100% of an organisations costs incurred over a given period are allocated to all the cost objects	Direct costs, variable overheads and fixed overheads are apportioned to cost objects. Sometimes required by financial reporting standards
Activity—based costing	A method of top-down micro-costing	Indirect expenditure is first allocated to tasks or activities, so that it can be apportioned to cost objects at a more detailed level of disaggregation than used in traditional top-down gross costing
Cost centre	Responsibility centre in an organisation where the cumulative operating expenses of a group of similar activities are recorded over a finite period of time.	E.g. a hospital laundry department cost centre might record the costs of staff and consumables used to operate the laundry service over a year. The cost centre would probably not include the costs of general hospital overheads such as maintenance of the building, or capital expenditure such as purchase of machinery
Cost pool (or activity cost pool)	Term used in Activity-Based Costing referring to an aggregate of all the (indirect and possibly overhead) costs associated with a particular task	In ABC, if a cost centres records expenditure related to multiple tasks, these tasks are first disaggregated to more detailed “activity cost pools” before being apportioned to cost objects using micro-data
Cost object (“output”)	Final product, process or service that are going to be costed	E.g. Hospital GRDs. Normally in top-down methods, all final services performed by the organisation during the accounting period will be costed
Activity cost driver/resource/activity	Measures that identify the linkage between indirect expenditure and cost objects. They serve as quantitative measures of the activity undertaken by cost centres	The costs of the laundry department might be allocated to cost objects in proportion with the number of days that patients spend in hospital (days in hospital is the activity cost driver for laundry department expenditure)
Activity cost driver rate (“unit cost”)	The amount determined by dividing the indirect activity expense by the total quantity of the activity cost driver	If the annual laundry department expenditure is 55.000€ and the laundry serviced 11.200 patient bed-days during the year, the activity cost driver rate will be 4.91€/day
Terms used in bottom-up costing		
Bottom-up (also known as variable costing or direct costing)	Cost components are valued by identifying resource use directly employed by each patient	Patient-specific costs
Cost object	Final product, process or service that are going to be costed	In bottom-up costing, usually only one cost object will be costed, e.g. cost of a specific surgical procedure and associated hospital stay
Resource	All materials, facilities, personnel, and anything else that is used for providing health care services	Medical, administrative and nurse staff, medical devices, health products, buildings, water, electricity, etc.
Unit cost	Refers to the marginal cost of providing a single unit of resource. Variable and sometimes fixed overheads are often approximately included by applying a percentage “mark-up” on direct cost, or by applying an average overhead “cost per day”	One hour of surgeon time, price of a dose of medication, etc.

### Top-down costing

Normally, the aim of top-down costing is to estimate mean costs for the full set of products and services (cost objects) produced by the organisation during a given period. The starting point is the actual cost of resources consumed by the organisation during a given period (usually obtained from retrospective data held in the organisation’s management accounting system or general ledger), which are assigned or apportioned down to cost objects (usually based on retrospective activity data held in the organisation’s administrative system over the same period).

Top-down costing is usually undertaken by the organisations themselves primarily for their own purposes (such as setting tariffs or financial reporting) using their own accounting data. This fact presents some challenges for economic evaluation. First, as mentioned in the introduction, financial accounts may not include all opportunity costs, e.g. if they are conducted on a cash basis. Some countries require healthcare providers to undertake full absorption costing to meet financial reporting requirements, such that all direct, variable overhead and fixed overhead costs are apportioned to all cost objects. Fixed overhead costs might include items such as amortisation of buildings, interest payments on loans, insurance, training of staff and general administration expenditure. These are fixed costs because they provide for the organisation’s current business operations, but do not increase or decrease with production levels unless considerable new capacity is required or the organisation closes. Whether or not these fixed costs should be included in an economic evaluation will depend on the research question and context. If there is spare capacity in the health system, then it may be reasonable to exclude these kinds of capacity-generating expenditures from economic evaluation. However, if the program under investigation has a sufficiently long time horizon and/or operates at a sufficiently large scale, then investment in new capacity will be required and these items should be taken into consideration. In any event, top-down costing generates mean costs, and these will only correspond to marginal costs under conditions of constant returns to scale.

If it is considered appropriate to include the opportunity costs of capital assets in economic evaluation, then there are two components of capital charges that ought to be taken into consideration. The first is to allow for the “consumption” or wearing out of the asset due to use or the passage of time. Organisations that follow IFRS will amortise the value of fixed assets such equipment or buildings in their accounts and periodically revalue their asset register to replacement value. However, land is non-depreciable as it retains its value over time [1, 7]. The second component of opportunity cost of capital assets is the alternative use of the land, buildings and other assets managed by the organisation. Public sector organisations in many countries treat capital assets as a free good, whereas private organisations are required to generate a return on capital employed that is paid as interest or dividend to investors. An exception is England, where since the 1990s NHS hospitals have been required to generate a return on assets (currently 3.5%), and this expense is included in their reported full absorption cost of services [8, 9]. This may be important for international comparison of costs in England with other countries which do not recognise these expenses.

A second challenge is the definition of the cost objects. For example, hospitals often use DRGs to define the set of products, processes and services to be costed. This system of classifying healthcare activity was originally developed for tariff setting rather than decision-making, and may lack the granularity required for economic evaluation of specific treatments or therapies. A third challenge is that by making use of historic (retrospective) data, top-down methods may not be able to estimate costs of new therapies which have not yet been widely used in clinical practice. Fourth, cost accounting methodologies require certain conventions and assumptions, which may differ across and within jurisdictions and institutions.

In top-down costing, costs can only be classified as “direct” (in a cost accounting sense) if the organisation collects information that enables the item of expenditure to be directly traced to a particular cost object (e.g. a specific DRG) [10]. Historically, few public healthcare organisations have had the administration or management systems to identify this level of detail. Exceptions might be some specific pharmaceuticals, or medical prostheses, that are only used for treating particular DRGs. In these cases, most of the organisation’s expenditure is treated as variable overhead (joint costs) or fixed overhead, and must be apportioned to cost objects using some proxy measure of activity (known as a cost driver) [11]. If a high proportion of costs have to be classified as overhead, then this is obviously a limitation of the top-down method, as any method of apportionment can only be an approximation.

Private healthcare organisations often have invested in more sophisticated accounting and information systems (because they need to bill patients or their insurers for items used) and in these cases can identify a greater proportion of costs directly to individual patients. Increasingly, public hospitals are also investing in their information systems, to improve management, provide better quality of care to patients, or meet the requirements of healthcare commissioners for case-based hospital payment [12]. One characteristic of top-down costing is that, although the method may be “inaccurate” (accuracy depending on the level of detail of organisational activity data to apportion overheads to cost objects) the method will be very “precise” in the sense that it allocates or apportions exactly 100% (no more and no less) of organisational expenditure incurred in a given period to the set of cost objects, and hence (unlike bottom-up) does not rely on sampling patients from the available population.

### Activity-based costing (ABC)

ABC was developed as a more accurate method of assigning overhead costs to final products, and as such can be classified “top-down micro-costing” [11, 13]. It requires the organisation to collect detailed data about each activity, typically gathered through interviews or direct observation of personnel by researcher [14–16].

The main difference between “traditional” top-down gross-costing and ABC is that ABC systems instead of using cost centres (accounting responsibility centres) for accumulating variable overhead costs, start by defining a set of “activities” performed by those resources [17]. Overhead costs are first allocated to activity centres, then traced to cost objects by multiplying the activity driver rate by the activity driver consumption [18, 19].

Time-driven activity-based costing (TDABC) is a simplified form of ABC. The difference between these two approaches lies in the type of the cost driver they use. A patient’s care cycle from the moment he is admitted to the hospital to the moment he is discharged may be very complex in terms of resources and time consumed. The traditional ABC uses a variety of cost drivers adapted to the specific situation (e.g. how many times an activity is conducted, size of space to be heated, etc.). However, the traditional ABC may be very difficult to implement [20]. TDABC only uses time as cost driver (e.g. machine hours, direct labour hours) [16]. The time required to perform an activity is likely to be driven by many time drivers. TDABC consists of applying a time equation that models how different time drivers drive the time devoted in activity. They can be included in one activity by means of time equations. Additionally, the TDABC has the ability to determine both the practical and the unused capacity [20].

### Bottom-up methods

Key feature of bottom-up is that resource data are collected for each individual patient, while in top-down, data are collected at the organisation level, e.g. cost centre. Bottom-up methods follow three phases—identification, measurement and valuation of resources—typically in that order. The researcher typically selects a sample of patients from a population and identifies a set of resources to be recorded during their care pathway for a determined period of time. The measurement of the resources will be in natural units: labour time of health workers, units of medicines, time use of room, items of medical equipment and so on [5]. The third step of the bottom-up methodology consists in assigning a monetary value to the resources used. This is usually carried out by multiplying the number of units of each resource used by a unit value (“unit cost”). For economic evaluation, this unit value should reflect the social opportunity cost of the resource use, although for pragmatic reasons many analyses take a partial—as opposed to a social—perspective [1].

For “primary resources”, such as labour time of health workers, or units of medicines, or use of a catheter, the most straightforward method is to use the price paid by the healthcare organisation to the supplier (the “market price”). Under theoretical conditions of perfect competition without externalities and other distortions, the market price of a resource will reflect its social opportunity cost. In practice, many health resources are not exchanged in markets, and most certainly not in perfectly competitive markets. For example, health workers’ salaries and prices of medicines are often set under national agreements, rather than by market forces [1].

More fundamentally in terms of cost accounting, many “resources” collected in bottom-up studies are, in fact, composites or bundles of various services. Examples of these are “time in hospital ward” or “radiograph”. However detailed and disaggregated the bottom-up micro-costing has been, the “cost” of a day in a hospital bed will ultimately include some resources which cannot be specifically traced to consumption by an individual patient, or are consumed jointly by several patients, that is, variable overheads (e.g. catering, laundry, supervision of the ward by nursing staff) and (depending on the research question) fixed overheads (e.g. lighting, maintenance, etc.). Likewise, the “cost” of a radiograph will be a composite of direct costs (e.g. film), variable overheads (e.g. apportioned technician’s time, apportioned machine power) and fixed overhead (e.g. amortised and apportioned capital cost of the equipment).

To value joint costs or composite resource units, the researcher is obliged either to return to “top-down” methods, or to use published tariffs or public prices of cost per day and so on. If “top-down” methods are chosen to value composite resources such as “day in hospital” then it needs to be recognised that these ought to exclude any direct costs which were recorded by the bottom-up data collection. For example, if the bottom-up data collection recorded the number of contact minutes that a patient was personally attended by a nurse, then this nursing cost should not be double-counted in the unit cost per day calculation. However, while the ward is being generally supervised by nursing staff on duty, the patient is sharing a variable overhead resource, the cost of which should be included in the unit cost per day. Such considerations mean that great care is needed to produce accurate calculation of unit costs of composite resources and the researcher cannot easily make use of standard unit cost estimates routinely produced by the organisation for its own purposes.

A tariff is the price paid by a public (or private) insurer to a health care provider for a certain service which is free or subsidised for the user [6]. Public prices are those paid to a public health insurance system by private/external users, which are not entitled to receive these services as regular beneficiaries. This could be, for instance, the case of tourists requiring health care services from the public system of the country they are visiting. So, for example, the researcher may value a radiograph using the public price charged by the hospital to tourists. Tariffs and public prices might not reflect the economic cost of the resources involved in the service concerned. In some organisations, they are not even calculated using a formal accounting method. However, they values might be considered appropriate for economic evaluation if the decision-maker takes a payer, rather than a societal perspective: it is the price the payer has to incur for a health service on behalf of a beneficiary.

Costing is time and effort consuming and, therefore, analysts need to decide how accurate and precise cost estimates need to be. Bottom-up methods require measuring the units of resources used in each alternative treatment. However, health economists do not always carry out specific calculations of the unit cost (monetary value) of the resources at the same institution as the resource use, but they rather “plug in” estimates available from existing unit cost databases (e.g. official price of medicines, national salary agreements, etc.). This approach may be less time consuming. A second advantage is that if the unit cost estimates were carefully compiled as an average of a representative sample of providers across the country, then they might be considered to have higher external validity and relevance for economic evaluation in that country than an institution-specific unit cost [21]. Third, if unit cost values are taken from a mandatory/official cost list, cost results would be more comparable between different studies, as it would be more difficult for analysts to intentionally bias the cost estimates to make a certain option look more cost-effective.

Due to the greater degree of control over the selection of patients to follow-up, and because a greater proportion of resources can be classified as “direct”, many authors consider bottom-up methods to be more accurate than top-down, and some go further by recommending bottom-up micro-costing as the “gold standard” for economic evaluation [5, 6, 22]. However, this general statement misses two evident points. First, if carefully implemented, bottom-up costing can accurately estimate individual resource use, but cannot (by definition) value composite resources, meaning that the researcher must return to proxy measures such as top-down costs and tariffs. Second, bottom-up is often based on following up resources used in a sample of patients, so the statistical precision of the method depends on the study sample size and may be further limited by dropout or missing data.

The previously defined costing methods are further described in the Supplementary Figs. 1–4.

## Methodology for the literature review

### Search strategy

The publications selected for the purpose of this study resulted from scoping review that followed the methodology of Arksey and O’Malley [23] and that was carried out to identify new approaches in costing methodologies in health care between 2005 and 2018. The aforementioned dates were chosen to update the literature review of costing methodologies made by Mogyorosy and Smith [6] that focused on publications between 1986 and 2005 [6]. The publications were identified through an electronic search of PubMed, Scopus and EconLit databases and the reference lists of the identified articles were examined. Grey literature sources such as OECD, European Commission, Google Scholar, NHS England, European Observatory on Health Systems and Policies, Department of Health (London), EUnetHTA, Centre of Health Economics (University of York) and World Bank were also searched. Only articles written in English or Spanish were included. The analysis of the included articles aims to provide a narrative overview of types of methods used in the costing literature. In the analysis, we apply the principle of saturation, that is, we focus on describing and classifying the diversity of methods employed rather than a detailed description of each article [23]. See Supplementary Table 1 for further details.

### Extraction of data

As an aid for conducting this review, we developed a checklist of qualitative questions that should be addressed by researchers when estimating costs for economic evaluation, based on the considerations outlined in “A general framework for classifying costing methods” of this study. As top-down and bottom-up methods are different in approach and information requirements, we developed a separate checklist for each. It is proposed that these checklists could be used more widely with the aim of improving the conduct and reporting of costing studies in the literature (Tables 3, 4).

**Table 3:** Checklist for methodology of bottom-up costing studies. Source: own elaboration

Bottom-up costing method Section	Justification/description/examples
Method of collecting resource use	
Selection of patients to follow-up	Illustrates how well patient group match research question (external validity)
Selection of number of patient	Provides information related to the precision of the study (internal validity)
Selection of period to follow-up	E.g. hospital episode, 1 year, etc.
Prospective/retrospective	Provides information about the accuracy of the study
Selection of resources to follow-up	Provides information about the accuracy of the study *Micro*-*costing:* resources are identified at very detailed level *Gross*-*costing:* resources are identified at highly aggregated level
Source of resource use data collection	Describes how were resources used collected. Provides information about the accuracy. E.g. electronic/administrative database, hospital notes, observation, questionnaire, interviews, etc.
Valuing resource use	
Method of valuing resource use	E.g. tariffs (public prices), hospital unit costs, national unit costs
Source of data for unit costs	Provides the institution responsible for calculating unit costs, the name of official source of unit costs, etc.
Method of estimating overheads	E.g. direct allocation, step-down allocation, step-down allocation with iterations, simultaneous allocation [1]
Variable overheads included	E.g. laundry, catering, maintenance, etc.
Fixed overheads included	E.g. amortisation of technology, amortisation of building, training and education, insurance, etc.
Analysis	
Handling missing data	Considers dropouts from prospective study
Handling of censored data	Considers inpatients that are not discharged before the study finishes

**Table 4:** Checklist for methodology of top-down costing studies. Source: own elaboration

Top-down costing method Section	Justification/description/examples
Study characteristics	
Design of the study	Single centre/multicentre
Type of centre	E.g. primary care centre, hospital, etc.
Purpose of the study	Provides type of cost that is going to be estimated. E.g. cost of primary resources, goods and services or processes and interventions
Level of detail in costing	
Identification of resources	Micro-costing/gross-costing
Cost object	Describes the final units that are going to be costed (e.g. GRDs, inpatient stay)
Direct costs	Provide a list of the types of costs that can be directly linked to each cost object (e.g. the medicines consumed by each patient during their hospital stay)
Indirect costs (variable overheads)	Provide a list of the types of costs that can only be indirectly linked to each cost object, but vary with the number of patients treated (e.g. labour costs, materials, laundry)
Indirect costs cost driver rate	Describes how is the activity used to link indirect costs to cost objects (e.g. cost of surgeon is measured by time spent on specific activity)
General overheads (fixed overheads)	Provide a list of costs that are considered non-patient care related (e.g. energy, insurance, R&D, land costs, etc.)
Overheads cost driver rate	Describes how is the activity used to link overheads to cost objects (e.g. cost of heating is distributed to direct costs by raising the direct costs with a mark-up percentage)
Data collection for activity or cost driver	
Prospective/retrospective	Provides information about the accuracy of the study (internal validity)
Source of resource use data collection	Describes how were resources used collected. Provides information about the accuracy. E.g. electronic/administrative database, hospital notes, observation, questionnaire, interviews, etc.
Source of costing data	Provides the institution responsible for reporting costs, the name of official source of unit costs, etc.

## Results

### Description of included papers

The following sections describe the methods used in the included literature according to whether they are top-down or bottom-up according to our glossary definition. If this differs from the author’s own classification then this is commented on.

**Fig. 1:**
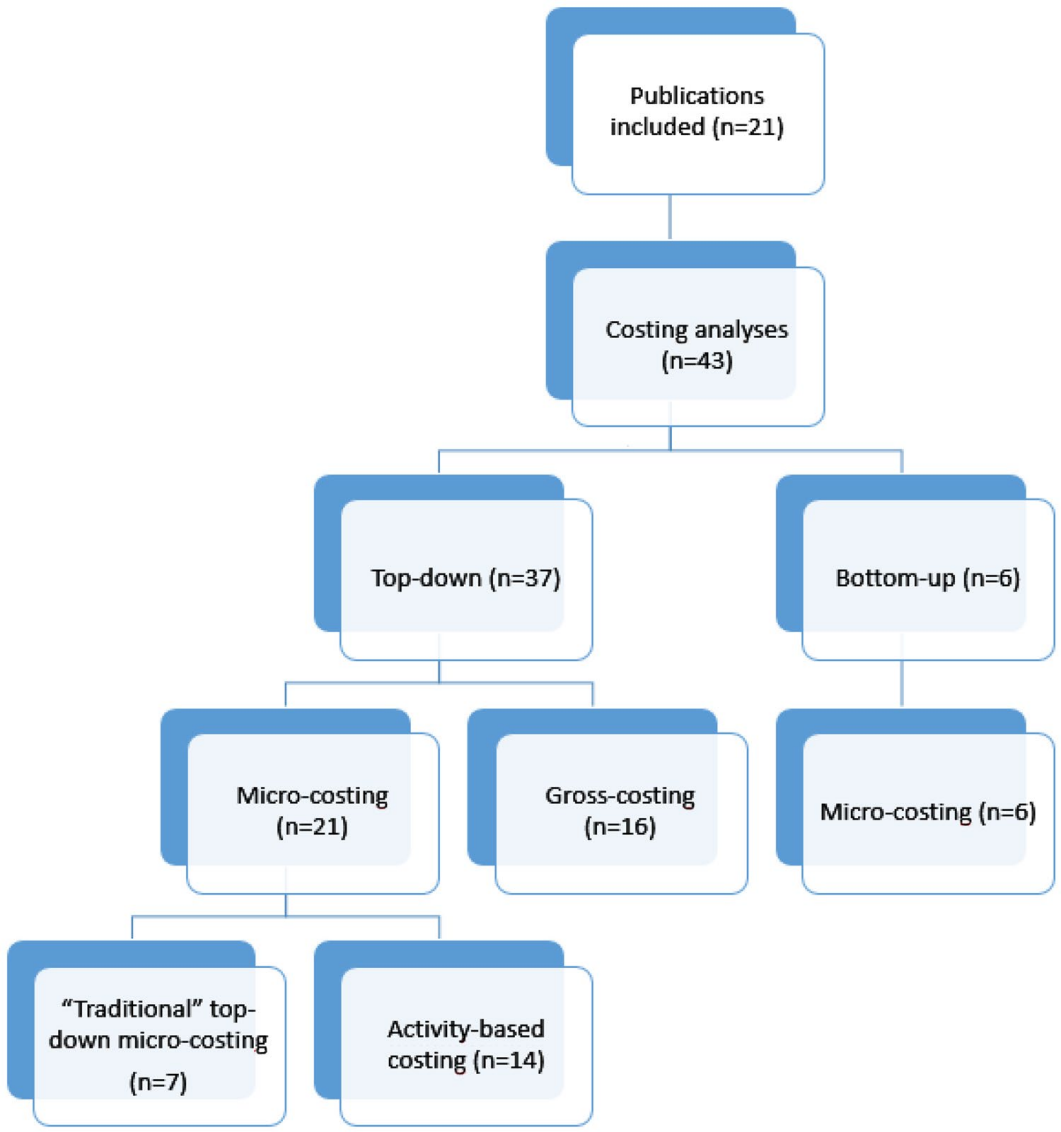
Description of included papers. Source: own elaboration

Twenty-one publications have been included in our analysis—eight of them have described one methodology and the remaining thirteen articles have focussed on the comparison of two or more different methodologies (Fig. 1). Therefore, 41 costing analysis have been included in total. Top-down micro-costing was the most common analysis (*n* = 21), followed by top-down gross-costing (*n *= 16) and bottom-up micro-costing (*n* = 4). No study could be described as “bottom-up gross-costing”. See Supplementary Fig. 5 for Prisma chart.

### Studies classified as using top-down gross costing

In some publications, several top-down gross-costing analyses have been performed. More detailed study characteristics are summarised in the Supplementary Table 2. Yarikkaya et al. [19] compared two methods. On the one hand, top-down gross-costing method, also known as volume-based costing or “traditional” costing system. Overheads were assigned to cost objects proportionally to the number of cost object (histopathological examinations). On the other hand, the estimates from the previous method were compared to national tariffs; however, the national tariffs were not based on any systematic costing methodology [19]. In Javid et al. [15], overheads were assigned to cost objects using patient-days as a cost driver [15]. Geue et al. [24] compared five top-down gross-costing analysis. The cost object was continuous inpatient stay in all cases, but the difference relied on what was the cost based on: health care resource groups (HRGs) (English tariff-setting method), HRGs (Scottish tariff-setting method), specialty and hospital-specific per diem cost, specialty and hospital-specific episode costs and individual length of stay (LOS), and specialty and hospital-specific episode costs and national average LOS [24]. In Chapko et al. [11], overheads were included in national budget. For that reason, they were assigned to cost object using same methods as direct costs [11]. In Clement et al. [25], the cost object is the number of inpatient; however, two different patient classifications are used. First, patients are classified into groups based on the most responsible diagnosis. To calculate the cost of each hospitalisation, each group is assigned a relative index value (that represents the complexity of each patient in comparison to the average patient) and multiplied by the average national cost per patient. The second methodology classified inpatients into groups based on the principal diagnosis. A weighted average of each group cost across hospitals is calculated and then adjusted for the severity of each case mix within hospitals [25]. Several authors [5, 10, 11, 15, 19, 21, 26] used volume-based allocation (such as, inpatient days, number of order receptions, intervention days or encounters, number of procedures, etc.) to assign overheads to cost objects.

In general, it is not clear what was included in overheads. Nevertheless, most of the publications included in this section used top-down gross-costing only to compare cost estimates with other analysis which was described in more detail. Resource use was collected from a hospital administrative database in three studies [11, 15, 24] and from national data set in two analyses [5, 21]. No mention about resource use data collection was done in remaining seven analyses [10, 19, 26, 27]. The most frequent collection of activity data was prospective [21, 24–26].

### Studies classified as using ABC or top-down micro-costing

Within studies classified as top-down micro-costing, the ABC was applied in 14 analyses [10, 14–16, 19, 26–32]. Out of them, simplified-ABC (consists of reducing the number of cost drivers, but maintains the same number of activities and cost objects) [10] and TDABC (consists of detailed list of administrative activities and the amount of time the medical and administrative staff spend on each patient) [16, 32] were applied in one and two analyses, respectively. Detailed ABC (that is, top-down micro-costing) was conducted in seven studies [5, 11, 14, 16, 22, 33]. In Hrifach et al. [22], once all resources have been identified, two different prices were placed on consumables to analyse differences in total cost of organ recovery program. This was described as two different “traditional” top-down micro-costing methods [22]. More information on these studies is given in Supplementary Table 2.

In general, the studies were characterised by large variety of level of detail in costing. In relation to the overheads, electricity, water and heating, capital equipment and depreciation, cleaning and maintenance costs were included in eight studies [5, 14, 15, 19, 26–28, 31] and financial costs (interest, etc.) were included in three studies [5, 14, 31]. Overheads were not disaggregated in detail in five publications [11, 16, 22, 30, 32] and overheads were not included in three studies [10, 29, 33]. In 11 of the studies, the type of cost driver used to trace indirect costs or overheads to cost objects was well defined (e.g. time for personnel costs, median size of the room used for space costs, cubic metres for water, kWh for electrical energy, etc.) [5, 10, 15, 19, 22, 26, 28, 30–33]. However, cost driver was missing in four publications [27, 29]. Unit cost sources were missing in almost all publications. Considering data collection tools, the combination of interviews, questionnaires, electronic database, direct observation or accounting reports was the most frequently used method [14, 15, 19, 22, 28, 31, 32], followed by direct observation [16, 33], questionnaires [29, 30] and electronic database [11] only. Nevertheless, four publications did not specify how resource use data were collected [5, 10, 26, 27]. Additionally, data were collected prospectively in eight publications [5, 26, 27, 29–33], retrospectively in three publications [11, 15, 22], both methods were used in two publications [16, 19] and this information was missing in three publications [10, 14, 28].

The findings in this review demonstrated that regarding top-down studies, there is a variety of resources considered as direct. In three studies, the resources are disaggregated into direct, indirect and overheads; however, no additional information about how direct costs were identified was provided [15, 16, 30]. In the other two studies, the information about whether the organisation collects information that enables the resources to be directly allocated to a particular cost object is missing [5, 29]. Therefore, it is not possible to properly judge whether the direct costs were defined according to the established cost-accounting terminology summarised in Table 2. Mercier and Naro [14] do not specify what resources are considered direct, indirect or overheads [14].

### Studies classified as using bottom-up methods

Four publications used bottom-up method to evaluate resource use [5, 21, 25, 34]. Individual participant case records and interviews were used to collect resource use data in two publications [21, 34], while the other two remaining publications did not provide this information [5, 25]. Resource use data were collected prospectively in all publications. Regarding method of valuing resource use, hospital unit costs were used in three publications [5, 25, 34]. In Olsson [21], three methods were used to estimate unit costs, first, equalling unit costs to the price paid for the intervention; second, estimating unit costs from total annual costs and workload measures; third, by taking the average of the available unit costs as calculated by one of the previously mentioned methods [21]. Overheads were allocated to direct costs in two studies by raising the direct costs with a mark-up percentage [5, 21]. While in Venkatnarayan et al. [34], the overheads taken into account were specified (energies, cleaning, capital equipment and depreciation) [34]; in three remaining articles, no mention about what type of overheads included was made [5, 21, 25]. More detail on these studies is given in the Supplementary Table 3.

## Discussion

### Summary of main findings

The results of this review show a considerable variability of terminology employed in costing studies as well as inappropriate use of the same. Unsurprisingly, it is not always clear what is meant by “direct” and “indirect” costs, as the common usage in the cost-accounting literature differs from that used in economic evaluation. Papers were not always clear about the distinction between top-down and gross-costing, and between bottom-up and micro-costing [6, 24, 25, 33]. Some studies described themselves as “ABC” when they might be more properly described as gross costing.

### Correct classification of top-down, bottom-up, gross-costing and micro-costing

In bottom-up costing, the researcher typically follows individual patients, collecting resources used by questionnaire or from hospital notes. These can be considered as “direct”. Hence, a greater proportion of total costs are usually classified as direct in bottom-up costing than in top-down. Nevertheless, bottom-up costing must still somehow account for all the other departmental and organisation-wide expenditure that have not been included in the individual patient follow-up. In no method can all costs be classified as direct. Overheads are shared resources used “simultaneously” by multiple patients. For that reason, it is usually infeasible or impossible to estimate the use of general utilities such as electricity, laundry, and water by a single patient. Any bottom-up costing method must address this challenge, but this is rarely recognised in the literature. An exception was Venkatnarayan et al. [34]. They estimated indirect costs by bottom-up micro-costing method (patients were followed in time) and overheads were identified by micro-costing and evaluated by top-down method (step-down or “cascade” method was used to place them to cost object). It is worth noting that, in the UK, there is currently an initiative to improve the accuracy and precision of cost accounting information by collecting detailed bottom-up data on resource use at patient level [35].

Other common confusions are the classification of the ABC as bottom-up method on the one hand and the top-down as bottom-up on the other hand. According to Mercier and Naro [14], “activity costs were calculated using cost drivers […]”. Nevertheless, there is no need to use cost drivers in the bottom-up costing studies, because all patients are followed in time and all resources consumed by each patient are attributed directly to the patients. Additionally, we suggest the bottom-up should be reserved for study of patients, because the patient is the ultimate cost object in costing exercise, and there was no follow-up of individual patients in this study. Therefore, both methods described in this article are top-down micro-costing. The real difference between them relies on cost driver rates. In top-down micro-costing, staff costs were traced to each procedure on basis of predetermined costs weights and remaining costs (drugs, materials and overhead costs) were traced proportionally to the indirect costs. In ABC micro-costing, the cost driver rates were based on the time administrative, nurse and medical staff dedicated to each activity.

Chapko et al. [11] describes a costing method that consists of assigning the workload for individual employees and costs from the general ledger to cost object. This method is described in the article as bottom-up. However, as we have previously demonstrated, the method based on using cost centres for accumulating costs and, subsequently, assigning them to indirect costs is top-down. Both methods used in Chapko et al. [11] are top-down. However, they differ in the way the overheads are allocated to final cost objects. The method that is mistakenly considered to be bottom-up uses different resource driver to allocate indirect costs and overheads, while in what was considered to be top-down method both according to the author and our classification, overheads are included in indirect costs and, therefore, they are allocated to cost objects in the same way as indirect costs [11].

Hrifach et al. [22] used micro-costing approach to estimate resource use. The estimation was based on discharge abstracts and discussion with hospital staff. All resources were valued by top-down approach using a hospital accounting reports. Additionally, the analyses were repeated valuing the consumables through the unit purchase price. This was classified by the authors as bottom-up approach. Notwithstanding, the only difference between these two analyses consisted of different valuation of one specific resource (consumables). Therefore, no bottom-up method was done in this publication [22].

Nevertheless, it is not clear how detailed the identification of resources should be to be considered micro-costing. Zarkin et al. [30] included labour costs, space costs, costs of laboratory tests, drug costs [30]. Ismail et al. [33] included costs of medical devices, labour costs, costs of re-usable and disposable instruments, but did not include space costs [33]. Rajabi [27] included costs of materials, labour costs, depreciation costs and overheads, but did not include space costs [27]. Clement et al. [25] included cost of nursing hours, the electricity need for lighting the recovery room, the catheter, the operator’s time, food costs, etc. [25]. Olsson [21] did not specify what costs have been considered when identifying resources at micro-costing level [21]. Therefore, to decide whether identification of resources is sufficiently detailed to be called micro-costing seems to be a matter of personal preference.

### Appropriateness of costing methods for economic evaluation

According to Drummond et al. [1], one of the crucial questions to pose in economic evaluation studies is the accuracy of costing. The strongest determinant is the alleged quantitative importance of each cost category included in the evaluation [1]. For instance, many laboratory tests cost only few cents each; therefore, it is not worthy to invest in cost estimation of each of them and the average laboratory charge is suffice. On the other hand, labour costs are often the largest component of final cost object [15, 26]. Therefore, the larger is the cost component in the total cost, the greater detail should be placed on its identification. Additionally, very detailed identification of resource use is especially useful for estimating the cost of innovative interventions, as they often do not have determined provider reimbursement rate or DRG weight, in other words, they have no pricing information available [36].

Other important issue to take into account while carrying out an economic analysis is the purpose and perspective of the study in question. Both of them will have considerable influence over the appropriateness of costing approach. Therefore, for local variation of the results, a bottom-up approach may be preferred; for generalization of the results over an entire service population, a top-down approach may be preferred [21].

When comparing accounting and economic analysis, several aspects should be considered. Organisations apply financial accounting and sometimes cost accounting for several reasons. Financial accounting is often mandatory for fiscal purposes; it is also necessary to assess the economic and financial situation and sustainability of the organisation, and to estimate the profits. Some organisations apply analytical/cost accounting to calculate the unit costs of the goods and services they produce; this is often the basis for price setting; comparing costs of alternative production technologies allows managers to identify the most efficient and profit maximising technologies. Economic evaluations try to assess the most cost-effective/efficiency options by comparing the (opportunity) costs, as well as the health consequences and health technologies, programmes and decisions compared. Resource costs are usually calculated by multiplying the number of units of a certain resource times a unit monetary value. The unit costs of health care organisations (usually an average which is valid/representative for the relevant setting) are normally used as a proxy of the unit costs estimated in economic evaluations. The concept of opportunity costs in EE includes but goes beyond financial and resource costs. On the other hand, valid accounting costs are sometimes not available for some resources and the economic evaluation must use other quantification approaches, such as market prices, shadow prices, tariffs, and contingent valuation.

Some of the reasons for which the aforementioned approaches diverge are summarised in Table 5.

**Table 5:** Differences between accounting cost and economic evaluation approach. Source: own elaboration

	Health service provider. Accounting cost	Health economist. Economic evaluation	Example
Source of data	Retrospective/historical data	Synthesis data from different sources to predict future costs of relevant treatment options	UK reference costs are published with 2-year lag
Objective	Financial reporting management	Opportunity cost of providing service	Hospitals are obliged to estimate costs using a specific methodology and using a standard report format
Time horizon and fixed costs	Short run total mean cost	Long run marginal cost	Providers may not distinguished between fixed and variable costs
Perspective	Single organizational perspective	System-wide or societal perspective	Providers only include items recorded in their accounting ledger. E.g., in some countries may not include depreciation or financing costs
Practical constraints	Lack of resources to undertake detailed micro-costing	Wish to include all relevant resources	Health service providers may not have the IT systems or personnel available to conduct detailed micro-costing

## Conclusion

The findings highlight the fact that no costing methodology can be considered as the gold standard. Bottom-up micro-costing method is very useful when aggregate costs are not available. Nevertheless, even if resource use is patient specific, there is a need to return to top-down methodologies to trace unit costs to cost items. The information regarding unit cost calculation is lacking. Additionally, it is not possible to calculate all costs using bottom-up method, because in case of overheads it is difficult to know the amount used by each patient and top-down method is needed to trace overheads to cost object. Therefore, convergence of bottom-up and top-down methods might be a hot topic for discussion in next decades. To improve accuracy and precision when estimating cost of an intervention, we offer the following suggestions.

1. Health economists to describe methods using a standard classification such as the one proposed in this article.
2. Providers to continue to invest in integrated IT systems that permit more precise understanding of the advantages performed in the organisation on the main drivers of resource use (e.g. PLICS).
3. Health systems to ensure that health organisations understand the full opportunity cost of the services they provide, in terms of the human and capital resources required.

## Electronic supplementary material

Below is the link to the electronic supplementary material.

Supplementary material 1 (DOCX 48 kb)

Supplementary material 2 (DOCX 11 kb)

Supplementary material 3 (DOCX 83 kb)

Supplementary material 4 (DOCX 47 kb)

Supplementary material 5 (DOCX 15 kb)
