# Correction to: A general framework for classifying costing methods for economic evaluation of health care

**DOI:** 10.1007/s10198-021-01313-0

**Published:** 2021-05-25

**Authors:** Zuzana Špacírová, David Epstein, Leticia García-Mochón, Joan Rovira, Antonio Olry de Labry Lima, Jaime Espín

**Affiliations:** 1 Andalusian School of Public Health/Escuela Andaluza de Salud Pública (EASP), Granada, Spain; 2 University of Granada, Granada, Spain; 3 CIBER en Epidemiología y Salud Pública (CIBERESP), Spain/CIBER of Epidemiology and Public Health (CIBERESP), Madrid, Spain; 4 Instituto de Investigación Biosanitaria ibs, Granada, Spain

## Correction to: The European Journal of Health Economics (2020) 21:529–542 10.1007/s10198-019-01157-9

The article A general framework for classifying costing methods for economic evaluation of health care, written by Zuzana Špacírová, David Epstein, Leticia García-Mochón, Joan Rovira, Antonio Olry de Labry Lima and Jaime Espín was originally published electronically on the publisher’s internet portal (currently SpringerLink) on 20rd January 2021 without open access. With the author(s)’ decision to opt for open choice, the copyright of the article changed on 16th March 2021 to © The Author(s) 2021 and the article is forthwith distributed under the terms of the Creative Commons Attribution 4.0 International License (http://creativecommons.org/licenses/by/4.0/), which permits use, duplication, adaptation, distribution and reproduction in any medium or format, as long as you give appropriate credit to the original author(s) and the source, provide a link to the Creative Commons license and indicate whether changes were made.

The original article has been corrected.

