# Supplementary material for: A general framework for classifying costing methods for economic evaluation of health care
Source: Eur J Health Econ. 2020 Jan 20;21(4):529–42. doi: 10.1007/s10198-019-01157-9 (PMC8149350; doi:10.1007/s10198-019-01157-9)
Supplement: Supplementary file 1 — Supplementary material 1 (DOCX 48 kb) [file 10198_2019_1157_MOESM1_ESM.docx]

Supplementary Figures

Supplementary Figure 1. Top-down gross-costing

Overhead activity pool (aggregated)

🡨All costs incurred during a given time period🡪

🡨All cateogories of expenditure incurred by the institution🡪

GRD 1

“Cost objects”

GRD 2

GRD 3

GRD 4

Cost driver

Activity measure

Cost / day (c)

🡨All activity associated with each cost object during a given time period🡪

🡨All activity undertaken by the institution🡪

Days GRD1 (W)

Days GRD2 (X)

Days GRD3 (Y)

Days GRD4 (Z)

Note: Top-down gross-costing calculates the average cost per day by dividing the total costs by total volume of activity measure.

Supplementary Figure 2. Top-down micro-costing

Overhead activity pools (disaggregated)

🡨All costs incurred during a given time period🡪

🡨All categories of expenditure incurred by the institution🡪

GRD 1

“Cost objects”

GRD 2

GRD 3

GRD 4

Cost drivers

Activity measures

Cost / day (c)

Number of drugs prescribed

Drugs GRD1 (M)

Drugs GRD2 (N)

Drugs GRD3 (O)

Drugs GRD4 (P)

🡨All activity associated with each cost object during a given time period🡪

Cost / drug (d)

Length of stay

Days GRD1 (W)

Days GRD2 (X)

Days GRD3 (Y)

Days GRD4 (Z)

Note: In top-down micro-costing, the total sum of costs to be allocated to cost centres remains the same as in the top-down gross-costing. Nevertheless, the activity is measured in more detail.

Supplementary Figure 3. Bottom-up gross-costing

Activity measure

🡨Resources used during a given time period🡪

🡨Resources used by the patients🡪

Cost

Patient 1

Patient 2

Patient 3

Patient 4

Unit cost (external)

Cost / day (c)

Length of stay

Patient 1

Patient 2

Patient 3

Patient 4

Cost per patient

Notes: In bottom-up gross-costing, patients are followed in time and therefore, the cost object (e.g., cost per diem, disease-specific cost per diem) should include all direct and overhead costs associated with the patient. Nevertheless, costs are estimated from a different source.

Supplementary Figure 4. Bottom-up micro-costing

Activity measure

🡨Resources used during a given time period🡪

🡨Resources used by the patients (as much detail as posible)🡪

Cost patient 1

Cost patient 2

Cost patient 3

Cost patient 4

Unit cost (external)

Cost / day (c)

Cost per patient

Days

Patient 1

Patient 2

Patient 3

Patient 4

Drug tyoe M

Patient 1

Patient 2

Patient 3

Patient 4

Cost / drug M (d)

Note: The resources used during a given time period are measured by each patient and cost per each patient is calculated.
