# Supplementary material for: A general framework for classifying costing methods for economic evaluation of health care
Source: Eur J Health Econ. 2020 Jan 20;21(4):529–42. doi: 10.1007/s10198-019-01157-9 (PMC8149350; doi:10.1007/s10198-019-01157-9)
Supplement: Supplementary file 2 — Supplementary material 2 (DOCX 11 kb) [file 10198_2019_1157_MOESM2_ESM.docx]

Supplementary Table 1. Search strategy

| #1 | Search "Costs and Cost Analysis/methods"[Mesh] |
| --- | --- |
| #2 | "Costs and Cost Analysis/methods"[Mesh] Filters: Publication date from 2005/01/01 |
| #3 | (("cost method*") OR ("costing method*")) |
| #4 | #2 OR #3 |
| #5 | ((((((((cost-effect* [Title]) OR "cost effect*"[Title]) OR Cost-benefit*[Title]) OR "Cost benefit*"[Title]) OR "Cost utility"[Title]) OR Cost-utility[Title]) OR Cost sav*[Title]) OR Cost-sav*[Title]) |
| #6 | #4 NOT #5 |
