# Supplementary figures and images for: A general framework for classifying costing methods for economic evaluation of health care
Source: Eur J Health Econ. 2020 Jan 20;21(4):529–42. doi: 10.1007/s10198-019-01157-9 (PMC8149350; doi:10.1007/s10198-019-01157-9)

Supplementary Figure 5. Prisma chart


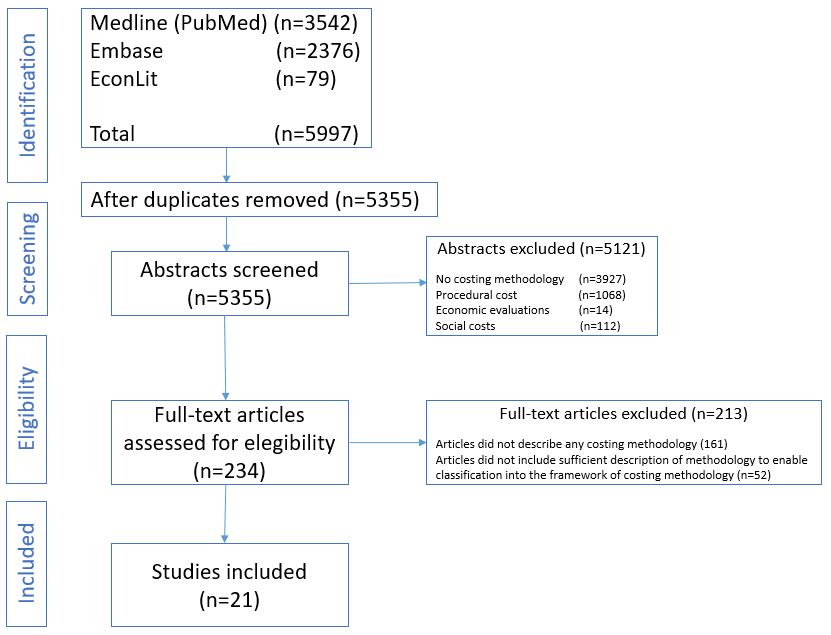


Source: Own elaboration

Supplement: Supplementary file 3 — Supplementary material 3 (DOCX 83 kb) [file 10198_2019_1157_MOESM3_ESM.docx]
