# Supplementary material for: A general framework for classifying costing methods for economic evaluation of health care
Source: Eur J Health Econ. 2020 Jan 20;21(4):529–42. doi: 10.1007/s10198-019-01157-9 (PMC8149350; doi:10.1007/s10198-019-01157-9)
Supplement: Supplementary file 4 — Supplementary material 4 (DOCX 47 kb) [file 10198_2019_1157_MOESM4_ESM.docx]

Supplementary Table 2. Characteristics of the top-down studies included in the analysis

| **Study characteristics** | | | | | | **Level of detail in costing** | | | | | **Data collection for activity or cost drivers** | | **Costing method** | |
| --- | --- | --- | --- | --- | --- | --- | --- | --- | --- | --- | --- | --- | --- | --- |
| **Author (Year of publication)** | **Year of data collection (if any)** | **Country** | | **Type of centre** | **Purpose of study** | **"Micro" or "gross"** | **"Cost objects"** | **Indirect costs** | **Overheads** | **Cost driver** | **Retrospective or prospective** | **Source of resource use data collection** | **According to the article** | **According to our classification** |
| Yarikkaya (2017) | 2014 (August 1-31) | Turkey | | hospital (SC) | cost of interventions | micro | average cost of intervention | materials |  | number of materials used | prospective | observation | ABC | ABC |
|  |  |  |  |  |  |  |  | personnel |  | working time | prospective | observation |  |  |
|  |  |  |  |  |  |  |  |  | electricity, water, natural gas | N/S | retrospective | electronic database |  |  |
|  |  |  |  |  |  |  |  |  | cleaning | N/S | retrospective | N/S |  |  |
|  |  |  |  |  |  |  |  |  | amortization of fixed assets | years of economic life (max 5 years) | retrospective | electronic database |  |  |
|  |  |  |  |  |  |  |  |  | amortization of the building | years of economic life (max 50 years) | retrospective | electronic database |  |  |
|  |  |  |  |  |  |  |  |  | stationery and consumables |  | retrospective |  |  |  |
|  |  |  |  |  |  |  |  |  | food |  |  |  |  |  |
|  |  |  |  |  |  | gross |  |  | overheads | fixed rate |  |  | VBC | VBC |
|  |  |  |  |  |  | gross |  |  |  |  |  |  | Tariffs | Tariffs |
| Hrifach (2016) | 2010 and 2011 | France | | hospital (SC) | cost of interventions | micro | total cost of organ recovery | medical and nursing staff |  | surgery relative cost index, anaesthesia RCI and a combination of the anaesthesia RCI and length of stay | retrospective | electronic database + interview | Top-down micro-costing | Top-down micro-costing |
|  |  |  | |  |  |  |  |  | logistics | isolating components specifically related to organ recovery | retrospective |  |  |  |
|  |  |  | |  |  |  |  |  | imaging | number or organs recovered from each donor | retrospective |  |  |  |
|  |  |  | |  |  |  |  |  | biology | number or organs recovered from each donor | retrospective |  |  |  |
|  |  |  | |  |  |  |  |  | consumables |  | retrospective |  |  |  |
|  |  |  | |  |  | micro |  |  | consumables |  | retrospective |  | Bottom-up micro | Top-down micro |
| Javid (2016) | 2012 | Iran | | hospital (SC) | cost of a service department | gross | cost of occupancy-bed day |  |  | patient-days |  | electronic database | TCS | TCS |
|  |  |  | |  |  | micro |  | personnel |  | time | retrospective | interviews | ABC | ABC |
|  |  |  | |  |  |  |  | materials |  | quantity of equipment | retrospective | interviews |  |  |
|  |  |  | |  |  |  |  |  | capital equipment and depretiation | strait-line depretiation approach | retrospective |  |  |  |
|  |  |  | |  |  |  |  |  | water, cleaning services, heating, indirect electricity | floor area | retrospective | accounting reports, annual expenditure report |  |  |
| Mercier (2014) | 2010 | France | | hospital (SC) | cost of intervention | micro | average cost per surgical procedure | medical, nurse, administrative staff |  | relative cost index | N/S | operating room register | Top-down micro-costing | Top-down micro-costing |
|  |  |  | |  |  |  |  | drugs, equipment | | allocated proportionally to the staff costs | N/S | operating room register |  |  |
|  |  |  | |  |  |  |  |  | supplies, taxes, insurance, utilities and loan interest | allocated proportionally to the staff costs | N/S | N/S |  |  |
|  |  |  | |  |  | micro | average cost per surgical procedure | medical, nurse, administrative staff |  | time spent on each activity |  | interviews | ABC bottom-up micro-costing | ABC |
|  |  |  | |  |  |  |  | drugs, equipment | | N/S |  | operating room register |  |  |
|  |  |  | |  |  |  |  |  | supplies, taxes, insurance, utilities and loan interest | N/S |  | N/S |  |  |
| Oker (2013) | 2007 | Cyprus | | hospital (SC) | cost of intervention (open and closed gallbladder operations) | micro | average cost per operation | personnel |  | directly allocated to cost object | prospective | observation | TCS | TCS |
|  |  |  | |  |  |  |  | drugs |  | directly allocated to cost object | prospective | observation |  |  |
|  |  |  | |  |  |  |  | room |  | directly allocated to cost object | prospective | observation |  |  |
|  |  |  | |  |  |  |  | operation and anaesthesia |  | directly allocated to cost object | prospective | observation |  |  |
|  |  |  | |  |  |  |  |  | overheads | inpatient-day | retrospective | observation |  |  |
|  |  |  | |  |  | micro | average cost per operation | personnel |  | time (practical capacity) | prospective | observation | TDABC | TDABC |
|  |  |  | |  |  |  |  |  | overheads | number of patients | prospective | observation |  |  |
|  |  |  | |  |  | micro | average cost per operation | personnel |  | time (ignores unused capacity) | prospective | observation | ABC | ABC |
|  |  |  | |  |  |  |  |  | overheads | number of patients | prospective | observation |  |  |
| Alrashdan (2012) | N/S | Jordan | | hospital (SC) | cost of intervention (cost of abdomen&pelvic CT procedure) | micro | average cost per patient per procedure per year | personnel |  | time | N/S | interviews, questionnaires, observations, motion and time study | ABC | ABC |
|  |  |  | |  |  |  |  |  | maintenance,accounting, administration, cleaning | amount of consumption, real data, area of activity location | N/S | accounting reports, estimation |  |  |
| Geue (2011) | 1972-2007 | Scotland | | hospital (N/S) | cost of intervention | gross | cost per continuous inpatient stay | N/S | N/S |  | prospective | hospital admission records | Top-down gross-costing | Top-down gross-costing |
|  |  |  | |  |  | gross | cost per continuous inpatient stay | N/S | N/S |  | prospective | hospital admission records | Top-down gross-costing | Top-down gross-costing |
|  |  |  | |  |  | gross | cost per continuous inpatient stay | N/S | N/S | specialty and hospital-specific per diem cost | prospective | hospital admission records | Top-down gross-costing | Top-down gross-costing |
|  |  |  | |  |  | gross | cost per continuous inpatient stay |  |  | specialty and hospital-specific episode costs and individual length of stay | prospective | hospital admission records | Top-down gross-costing | Top-down gross-costing |
|  |  |  | |  |  | gross | cost per continuous inpatient stay | N/S | N/S | specialty and hospital-specific episode costs and national average length of stay | prospective | hospital admission records | Top-down gross-costing | Top-down gross-costing |
| Olsson (2011) | 2004-2007 (2 year period for each patient) | Sweden | | Social welfare administrations (MC) | cost of intervention | gross | total cost per intervention | yes |  | intervention day or encounter | prospective | individual participant case records (hospital database) | Top-down gross-costing | Top-down gross-costing |
|  |  |  | |  |  |  |  |  | yes | intervention day or encounter | N/S | included in national data set |  |  |
| Chapko (2009) | N/S | USA | | Outpatients' clinics (MC) | cost of intervention | gross | outpatient encounter, individual patient, inpatient admission, particular services, DRGs | N/S | ambulatory care | medical resource based relative value scale | retrospective | electronic medical records | Top-down gross-costing | Top-down gross-costing |
|  |  |  | |  |  |  |  |  | hospital care | number of inpatients | retrospective | electronic medical records |  |  |
|  |  |  | |  |  |  |  |  | long-term care | resource utilization group and length of stay | retrospective | electronic medical records |  |  |
|  |  |  | |  |  | micro | outpatient encounter, individual patient, inpatient admission, particular services, DRGs | personnel, equipment, supplies |  | RVU | retrospective | electronic medical records | Bottom-up micro-costing | Top-down micro-costing |
|  |  |  | |  |  |  |  |  | yes | step-down method based upon either direct costs or occupied space | retrospective |  |  |  |
| Clement (2009) | 1995-2001 | Canada | | hospital (MC) | cost of intervention | gross (refined-grouper number) | average inpatient cost | N/S | N/S | weighted average of each RGN cost is calculated and then adjusted for the severity of case mixes within hospitals | prospective | N/S | Top-down gross-costing | Top-down gross-costing |
|  |  |  | |  |  | gross (case-mix groupers) | average inpatient cost | N/S | N/S | relative index weight | prospective | N/S | Top-down gross-costing | Top-down gross-costing |
| Yen Ju Lin (2007) | 2001 (June-September) | Taiwan | | hospital (SC) | cost of service lines of colorectal surgery department | micro | total cost per service lines | N/S | N/S | N/S | prospective | activities self-recorded by all personnel (questionnaires) | ABC | ABC |
| Cao (2006) | N/S | Japan | | hospital (SC) | cost of goods and services (laboratory tests) | gross | total costs per lab tests | yes | yes | annual total number of order reception | N/S | N/S | TCS | TCS |
|  |  |  | |  |  | micro | total costs per lab tests | yes | no | several cost drivers (nº of bills, nº of tests, time) | N/S | N/S | ABC | ABC |
|  |  |  | |  |  | micro | total costs per lab tests | yes | no | several cost drivers (nº of bills, nº of tests, time) | N/S | N/S | S-ABC | S-ABC |
| Suthummanon (2005) | 2001 (January-December) | USA | | hospital (SC) | cost of services (bone scan, muga scan, cardiac SPECT, PET scan, renal scan, thyroid scan) | micro | total cost per procedure |  |  |  | prospective | N/S | ABC | ABC |
|  |  |  | |  |  |  |  | personnel |  | time |  |  |  |  |
|  |  |  | |  |  |  |  | machine |  | time |  |  |  |  |
|  |  |  | |  |  |  |  |  | recruitment, employee training, physical plant | time |  |  |  |  |
|  |  |  | |  |  |  |  |  | office supplies, telephone, UM computer services | nº of procedures |  |  |  |  |
|  |  |  | |  |  | gross | average cost per procedure (assumes that all procedures are alike) |  |  | nº of procedures | prospective | N/S | TCS | TCS |
| Tan (2009) | 2005 | The Netherlands | | hospital (MC) | cost of intervention | micro | total cost of intervention |  |  |  |  |  | Top-down micro | Top-down micro |
|  |  |  | |  |  |  |  | imaging, drugs, labour, inpatient stay (hotel and nutrition, devices |  | norm-time (because now resource use is available only for average patient) | prospective | N/S |  |  |
|  |  |  | |  |  |  |  |  | general expenses, administration and registration, energy, maintenance, insurance, personnel costs of supportive departments | m2, staff full time units (several cost drivers) | N/S | N/S |  |  |
|  |  |  | |  |  | gross | total cost of intervention | inpatient stay (hotel and nutrition) |  | inpatient-day | retrospective |  | Top-down gross-costing | Top-down gross-costing |
|  |  |  | |  |  |  |  |  | general expenses, administration and registration, energy, maintenance, insurance, personnel costs of supportive departments | inpatient-day | retrospective |  |  |  |
| Demeere (2009) | N/S | Belgium | | outpatient clinic (SC) | cost of process | micro | average cost per consultation | labour, machine, material, cost of cabinets | N/S | time | prospective | direct observation + interviews | TDABC | TDABC |
| Zarkin (2009) | N/S | USA | | (MC) | cost of process | micro | mean cost per patient | personnel |  | time | prospective | questionnaires | ABC | ABC |
|  |  |  | |  |  |  |  | lab tests |  | time | prospective | questionnaires |  |  |
|  |  |  | |  |  |  |  | materials |  | time |  |  |  |  |
|  |  |  | |  |  |  |  |  | space costs | median size of the room used | prospective |  |  |  |
| Carvalho Jericó (2009) | 2006 | Brazil | | hospital (SC) | cost of process | micro | mean cost per patient |  |  |  | prospective | documentary research techniques + non participant closed observation | ABC | ABC |
|  |  |  | |  |  |  |  | personnel |  | time |  |  |  |  |
|  |  |  | |  |  |  |  |  | water | m3 |  |  |  |  |
|  |  |  | |  |  |  |  |  | building depreciation, hygiene and janitorial services, insurance, installation maintenance | m2, staff full time units (several cost drivers) |  |  |  |  |
|  |  |  | |  |  |  |  |  | electrical energy | KWh |  |  |  |  |
| Rajabi (2008) | 2005 | Iran | | hospital (SC) | cost of service | micro | cost per bed day |  |  |  | prospective |  | ABC | ABC |
|  |  |  | |  |  |  |  | personnel |  |  |  | N/S |  |  |
|  |  |  | |  |  |  |  | materials |  |  |  | N/S |  |  |
|  |  |  | |  |  |  |  |  | equipment depreciation |  |  | N/S |  |  |
|  |  |  | |  |  |  |  |  | kitchen centre |  |  | N/S |  |  |
|  |  |  | |  |  | gross | N/S | N/S | N/S | N/S | N/S | N/S | Fixed tariffs | Fixed tariffs |
| Ismail (2015) | 2012 | France | | hospital (SC) | cost of intervention | micro | cost per operation |  |  |  |  |  | Top-down micro | Top-down micro |
|  |  |  | |  |  |  |  | personnel |  | time |  | direct observation |  |  |
|  |  |  | |  |  |  |  | medical device |  | nº of operations+life expectancy |  | direct observation |  |  |
|  |  |  | |  |  |  |  | re-usable instruments |  | max nº of uses allowed |  | direct observation |  |  |
| Source: Own elaboration | | |  |  |  |  |  |  |  |  |  |  |  |  |
| Notes: SC: single centre; MC: multicentre; VBC, volume-based costing; TCS, traditional costing system; ABC, activity-based costing; S-ABC, simplified ABC | | | | | | | | | | | |  |  |  |
