# Supplementary material for: A general framework for classifying costing methods for economic evaluation of health care
Source: Eur J Health Econ. 2020 Jan 20;21(4):529–42. doi: 10.1007/s10198-019-01157-9 (PMC8149350; doi:10.1007/s10198-019-01157-9)
Supplement: Supplementary file 5 — Supplementary material 5 (DOCX 15 kb) [file 10198_2019_1157_MOESM5_ESM.docx]

Supplementary Table 3. Characteristics of bottom-up studies included in the analysis

| **Study characteristics** | | | | | | **Method of collecting resource use** | | | | | **Valuing resource use** | | | |
| --- | --- | --- | --- | --- | --- | --- | --- | --- | --- | --- | --- | --- | --- | --- |
| **Author (Year of publication)** | **Year of data collection (if any)** | **Country** | **Type of centre** | **Purpose of study** | | **"Micro" or "gross"** | **Length of follow-up (e.g., hospital episode, 1 year, etc.)** | **Source of resource use data collection** | **Retrospective or prospective** | **How were patients selected for inclusion in the study?** | **Method of valuing resource use** | **Method of estimating overheads** | **"Utility" overheads included** | **"Capital" overheads included** |
| Olsson (2011) | 2004-2007 | Sweden | Social welfare administrations (MC) | | cost of intervention | case-mix group | 2 years each participant | individual participant case records | prospective | patients aged 12-17 who fulfilled the criteria for a clinical diagnosis of conduct disorder according to the DSM-IV-TR | price (paid for intervention) | % mark-up over direct costs | N/S | N/S |
|  |  |  |  |  |  | micro |  |  |  |  | 75% of the total operating costs |  |  |  |
|  |  |  |  |  |  | case-mix group |  |  |  |  | average cost |  |  |  |
| Clement (2009) | 1995-2001 | Canada | hospital (MC) | | cost of intervention | micro | 4 year (each patient) | N/S | prospective | patients undergoing stented percutaneous coronary intervention from 1995 to 1997 resident in Calgary Health Region | hospital unit costs | N/S | N/S | N/S |
| Tan (2009) | 2005 | The Netherlands | hospital (MC) | | cost of intervention | micro | 1 year | N/S | prospective | N/S | hospital unit costs (diagnostics and devices-from financial hospital databases, drug costs-hospital pharmacies, labour costs-labour agreements, inpatient stay-annual accounts of 2005, overheads-annual accounts of 2005) | marginal mark-up allocation (mark-up was determined by dividing annual indirect costs by annual direct costs) | general expenses, administration and registration, energy, maintenance, personnel costs of supportive departments | insurance |
| Venkatnarayan (2014) | 2010 (October 2010-January 2011) | India | hospital (SC) | | cost of service unit | micro | 3 months | interviews | prospective | infants admitted to ICU within 24 hours of life | hospital unit costs | N/S | laundry, electricity, general stores, stationary stores, equipments of general use | land, fixed assets, amortization |
| Source: Own elaboration | |  |  | |  |  |  |  |  |  |  |  |  |  |
| Notes: SC, single centre; MC, multicentre | | | | |  |  |  |  |  |  |  |  |  |  |
